# Obesity and Prostate Cancer: The Tip of a High Mountain Still to Be Conquered

**DOI:** 10.3390/jcm9072070

**Published:** 2020-07-01

**Authors:** Cosimo De Nunzio, Vincenzo Ficarra, Andrea Tubaro, Giacomo Novara

**Affiliations:** 1 Department of Urology, Ospedale Sant’Andrea, Sapienza University of Rome, via di Grottarossa 1035-1039, 00189 Rome, Italy; cosimodenunzio@virgilio.it (C.D.N.); andrea.tubaro@uniroma1.it (A.T.); 2 Department of Human and Pediatric Pathology “Gaetano Barresi”, Urologic Section, University of Messina, Via Consolare Valeria, 98125 Messina, Italy; vficarra@unime.it; 3 Department of Surgery, Oncology, and Gastroenterology, Urology Clinic, University of Padua, Via Giustiniani 2, 35100 Padua, Italy

The narrative review of Fujita K et al. [1] addresses a significant although not well-defined issue: the relationship between obesity, inflammation, and prostate cancer. The authors should be congratulated for again focusing attention on such an essential but still not wholly understood topic. Prostate cancer (PCa) is the second most frequently diagnosed cancer, and age, race, and family history are the only established risk factors associated with prostate cancer development. However, the considerable geographic variation in prostate cancer risk suggests that lifestyle factors, such as physical activity, and a higher intake of dietary fat and meat may play a significant role in the pathogenesis of prostate cancer. Buschemeyer III WC and Freedland SJ firstly summarized, in 2007, the possible relationship between obesity and prostate cancer and the possible mechanism behind this association [2]. Specifically, they reported that obese patients presented an increased risk of aggressive prostate cancer, a larger prostate volume, a lower prostate specific antigen (PSA) value, more difficult surgery, and a poor outcome when compared to non-obese patients. The authors also postulated the limits of the body mass index definition of obesity and the importance of more precise measures of obesity, such as waist circumference, waist-to-hip ratio and percent body fat. Hormone changes, a poor diet, insulin resistance, and prostatic inflammation have generally been considered the critical pathological factors explaining the relationship between obesity and prostate cancer. De Nunzio and co-workers [3,4] also summarized the role of prostatic inflammation in the development and progression of prostatic diseases. The prostate is considered an immunocompetent organ, with a complex intraglandular immune system, defined as the prostate-associated lymphoid tissue (PALT). That ensures the sterility of the genitourinary tract and the prevention of autoimmune reactions towards self-antigens (XX). Several stimuli that activate different molecular pathways have been described as triggers for the dysregulation of the PALT and the development of inflammatory infiltrates, which are a common finding in biopsies and surgical specimens of prostate tissue from patients with prostate cancer or benign prostatic hyperplasia. Fujita K et al. [1] updated and improved this analysis including further possible molecular mechanisms including the role and distribution of several cells involved in the immunocompetent response, such as macrophages, myeloid-derived suppressor cells, B cells, and neutrophils. Additionally, the authors propose a role for the intestinal microbiome as a possible key factor in the relationship between obesity and prostate cancer. They also postulated that, as observed for colon and liver cancer, obesity and a high-fat diet could change the intestinal microbiome, which may play an essential role in prostate health and disease [5]. Unfortunately, most of these pieces of evidence are still preliminary and mostly related to in vitro or in vivo studies, while few data are coming from clinical trials. To date, an analysis of the peer-reviewed literature highlights several problems concerning this topic. Firstly, there is a paucity of prospective studies evaluating the role of diet, metabolic factors, and physical activity on inflammation and prostate cancer development and progression. Moreover, essential studies should address the possible effect of lifestyle changes such as weight loss and increased physical activity on the reversal of prostate cancer’s aggressive characteristics. Thus, the prevention and treatment of obesity could reduce prostatic inflammation, resulting, therefore, in the amelioration of prostate cancer development and progression or a better outcome after treatment. The first-line treatments for obesity indeed consist of lifestyle modifications, including an improved diet, weight loss, and increased physical activity. 

The lack of accurate and clinically available biomarkers of prostatic inflammation also represent a significant limitation of all the studies in this field. To date, the role of inflammatory mediators as new possible therapeutic targets for prostate cancer management and prevention cannot be accurately and clinically proved. The definition of obesity and the role of evaluating a single metabolic factor alone are still an open debate. Obesity is often part of a more complex disorder known as metabolic syndrome (MetS). MetS—a worldwide epidemic cluster of several metabolic abnormalities including hypertension, obesity, dyslipidemia, and insulin resistance—represents a well-known risk factor for coronary artery disease, cardiovascular atherosclerotic diseases, and diabetes mellitus type 2. MetS resulting from a Western diet and sedentary lifestyle is also associated with increased PCa incidence, aggressiveness, and mortality [6]. In the literature, the different components of MetS, such as obesity, have been individually observed to be directly associated with PCa risk. It is evident that the evaluation of PCa risk is complicated, because the different combinations of the various metabolic abnormalities that define the presence of the syndrome may influence PCa risk differently, positively or negatively. It has also been suggested that evaluating the single components of the syndrome may confound or obscure a comprehensive assessment of the patient’s metabolic status. Therefore, it has been suggested that further basic and clinical studies should be performed to evaluate this association by investigating all of these metabolic conditions as a whole, and they should also include an evaluation of the lifestyle factors such as diet and physical activity that have a significant impact on the syndrome [6]. A more comprehensive evaluation of all the possible different metabolic and lifestyle factors involved in prostate health together should help to better elucidate the complex molecular mechanisms behind these associations and better determine the possible clinical implications for prevention and treatment. Unfortunately, two decades of research in this field have only revealed the tip of a high mountain that, someday, someone will conquer. 
